# The Effect of Adherence to Mediterranean Diet on Disease Activity in Patients with Inflammatory Bowel Disease

**DOI:** 10.5152/tjg.2023.22193

**Published:** 2023-07-01

**Authors:** Kevser Çelik, Hakan Güveli, Yusuf Ziya Erzin, Emre Batuhan Kenger, Tuğçe Özlü

**Affiliations:** 1 Department of Nutrition and Dietetics, Bahçeşehir University Faculty of Health Sciences, İstanbul, Turkey; 2 Department of Gastroenterology, İstanbul University Cerrahpaşa Faculty of Medicine, İstanbul, Turkey

**Keywords:** Crohn’s disease, inflammatory bowel disease, Mediterranean diet, nutrition, ulcerative colitis

## Abstract

**Background/Aims:**

Mediterranean diet, owing to its inflammation-modulatory effects, is considered a beneficial dietary regimen for patients with inflammatory bowel disease. Despite promising results in the literature, studies on this subject are still limited. Therefore, the aim of this study was to evaluate adherence to the Mediterranean diet in patients with inflammatory bowel disease and examine its impact on disease activity and quality of life.

**Materials and Methods:**

A total of 83 patients were included in the study. Mediterranean Diet Adherence Scale was used to evaluate adherence to the Mediterranean diet. Crohn’s Disease Activity Index was used to evaluate disease activity in Crohn’s disease. Disease activity was determined by using the Mayo Clinic score for ulcerative colitis. Quality of Life Scale Short Form-36 was used to evaluate the quality of life of patients.

**Results:**

When the median Mediterranean Diet Adherence Scale score was 7 (1-12), only 18 patients (21.7%) showed strong adherence to the Mediterranean diet. Disease activity scores of patients with ulcerative colitis having low adherence to the Mediterranean diet were found to be higher (*P* < .05). In addition, some quality of life parameters were relatively higher in patients with ulcerative colitis who showed strong adherence to the Mediterranean diet (*P* < .05). For Crohn’s disease, no significant difference was found in disease activity and quality of life with respect to adherence to the Mediterranean diet (*P* > .05).

**Conclusion:**

Stronger adherence to the Mediterranean diet in patients with ulcerative colitis can help improve quality of life and modulate disease activity. However, further prospective studies are needed to investigate the potential use of the Mediterranean diet in inflammatory bowel disease management.

## INTRODUCTION

Inflammatory bowel diseases (IBDs) have recently emerged as a public health problem with increasing prevalence and severity worldwide.^1^ Inflammatory bowel disease, which includes Crohn’s disease (CD) and ulcerative colitis (UC), is an autoimmune disorder that usually begins at a young age and results in chronic inflammation of the gastrointestinal tract that persists throughout life.^2^ The IBD is more common in societies with high socioeconomic levels, and the highest incidence rates are observed in Western Europe and North America. The mean incidence and prevalence rates in Western Europe and North America for CD are 6/100,000 and 150/100,000 and those for UC are 20/100,000 and 200/100,000, respectively.^3^ In a multicenter study conducted in Turkey, the incidence of UC and CD in the population was 4.4/100,000 and 2.2/100,000, respectively.^4^

Although the etiological factors of IBD are unclear, it is thought to be caused by interactions between genetic factors, gut microbiome, and environmental factors.^5^ The dominant role of the intestinal microbiota in IBD is attributable to microbial dysbiosis, which includes a decrease in microbial diversity and an increase in pathogenic microorganisms.^6^ In genetically predisposed individuals, the dysbiotic microbiota are triggered by environmental factors, especially dietary components, and induce an immune response and affect the course of IBD.^5,7^

Although nutrition has an important role in the development and management of IBD, current studies on the role of diet in this disease are limited. Dietary components with pro-inflammatory potential are believed to cause changes in the immune system and intestinal microbiota, trigger damage to the mucous membrane, and cause IBD-related lesions.^8^ The Mediterranean diet (MD) is one of the most comprehensively examined dietary models in terms of its effect on chronic diseases.^9^ Evidence suggests that greater adherence to MD is associated with a significant reduction in cardiovascular diseases, cancer, and neurodegenerative diseases.^10-12^ Recent studies have reported new and promising data on the effects of MD on IBD.^13,14^ This effect is thought to be due to the role of some nutritional elements in MD, such as fiber, short-chain fatty acids, omega-3, polyphenols, and antioxidants, in regulating inflammation.^8^ In addition, MD has positive effects on quality of life as well as protective effects against diseases.^15^ In parallel with this finding, it becomes even more important to determine adherence to MD in patients with IBD with low quality of life scores compared to healthy controls.^16^ Considering the potential relationship between IBD and nutrition, the purpose of the present study was to examine the effect of adherence to MD on disease activity and quality of life in patients with IBD.

## MATERIALS AND METHODS

### Study Population and Sample

This cross-sectional study was performed between April 2021 and June 2021 at the Gastroenterology outpatient clinic of İstanbul University Cerrahpaşa Medical Faculty Hospital. Power analysis was performed to calculate the sample size. Based on the variables and group difference analyses, the minimum sample size was calculated as 60. A total of 83 volunteer patients (age, ≥18 years), who were diagnosed with IBD by the gastroenterologist (38 UC and 45 CD patients) and who were attending routine check-ups, were included in the study. Patients younger than 18 years; patients with perception disorders, psychological, and communication problems; and patients who did not volunteer to participate in the study were excluded.

At the beginning of the study, a questionnaire on sociodemographic characteristics such as age, educational status, anthropometric measurements, cigarette-alcohol consumption, year of diagnosis, and type of diagnosis was applied by the researcher during face-to-face interviews. All participants who volunteered to participate in the study were informed about the study verbally and in writing. Ethical approval was obtained by the ethics committee of Bahçeşehir University (no: 2021-04/01). The study was conducted in accordance with the principles of the Declaration of Helsinki.

### Determination of Disease Activity

Disease activity of the participants was determined by the physician at Cerrahpaşa Medical Faculty Hospital in İstanbul. Crohn’s Disease Activity Index (CDAI) was used to evaluate disease activity in CD. Disease activity was determined via Mayo Clinic score for UC. For CD, CDAI scores of <150, 150-219, 220-450, and >450 indicated remission, mild disease, moderate disease, and severe disease activity, respectively.^17^ For UC, Mayo Clinic scores of 0-2, 3-5, 6-10, and 11-12 indicated remission, mild disease, moderate disease, and severe disease activity, respectively.^18^

### Evaluation of Adherence to Mediterranean Diet

Adherence to MD of patients was assessed by face-to-face interviews conducted by a dietician (KC). Mediterranean Diet Adherence Scale (MEDAS) was used to evaluate adherence to MD.^19^ Pehlivanoglu et al^20^ reported that the Turkish version of the scale was reliable. The scale consists of 14 items; a score of 1 or 0 is given for each question according to the amount of consumption and the total score is calculated. The total score that can be obtained from the scale varies between 0 and 14. Low scores indicate low adherence to the MD and vice versa. The MEDAS scores of ≤6, 7-9, and ≥9 are categorized as low, acceptable, and high adherence to MD, respectively.^19^

### Evaluation of Quality of Life

Quality of Life Scale Short Form-36 (SF-36) developed by Ware and Sherbourne^21^ was used to assess the quality of life of patients. The reliability and validity study of the Turkish version of the SF-36 was carried out by Kocyigit et al.^22^ The scale consists of 36 items and provides a measurement of the following 8 dimensions: physical function (10 items), social function (2 items), role limitations due to physical functions (4 items), role limitations due to emotional problems (3 items), mental health (5 items), energy/vitality (4 items), pain (2 items), and overall health perception (5 items). The total score is not calculated for SF-36. Each dimension is scored and evaluated separately. Each dimension is assessed between 0 and 100 points. Low scores indicate poor health conditions, whereas high scores indicate good health conditions.^21^

### Statistical Analysis

Statistical analysis of the data was performed using the Statistical Package for the Social Sciences software (version 21.0) (IBM Corp. Armonk, NY, USA) package program. Statistical significance was indicated by *P* < .05 in all analyses. Descriptive statistics were presented as number, percentage, mean, and standard deviation. The conformity of data to normal distribution was checked by Kolmogorov–Smirnov and Shapiro–Wilk tests. Chi-square test was used to examine qualitative variables. Since the data were not distributed normally, non-parametric methods were used in the analyses. Mann–Whitney *U*-test and Kruskal–Wallis test were used to evaluate intergroup differences. Spearman analysis was used to evaluate correlations between continuous variables.

## RESULTS

Among the 83 patients (mean age, 37.4 ± 11.8 years) with IBD included in the study, 38 and 45 were diagnosed with UC and CD, respectively. The median CDAI score of patients with CD was 134 (7-416), whereas the median Mayo Clinic score of patients with UC was 5 (1-12). Although the majority of patients with CD were in remission (60%), 47.4% of the patients with UC presented with mild disease. The median MEDAS score of the participants was 7 (1-12). The majority of the patients (45.8%) showed low adherence to MD. The descriptive characteristics of the participants are shown in Table 1.

Table 2 shows the relationship between disease activity scores and BMI of the patients and adherence to MD. The UC patients with low adherence to MD had higher Mayo Clinic scores (*P* < .05). In contrast, no significant difference was found in CDAI score and BMI with respect to adherence to MD (*P* > .05).

Table 3 shows the correlation between disease activity scores and quality of life sub-dimension scores measured by SF-36. A significant negative correlation was found between CDAI score and role limitations due to physical functions (*r* = −0.290, *P* = .011), pain (*r* = −0.260, *P* = .016), and overall health (*r* = −0.283, *P* = .008). In addition, a significant negative correlation was found between the UC Mayo Clinic score and role limitations due to physical functions (*r* = −0.324, *P* = .014), role limitations due to emotional problems (*r* = −0.290, *P* = .026), energy/vitality (*r* = −0.332, *P* = .006), mental health (*r* = −0.285, *P* = .018), and overall health perception (*r* = −0.362, *P* = .003).

Table 4 shows the relationship between adherence to MD in patients with CD and quality of life. In patients with UC, those with higher adherence to MD had higher scores in role limitations due to emotional problems, mental health, and overall health perception (*P* < .05). In patients with CD, no significant difference was found in SF-36 sub-dimension scores with respect to MD adherence (*P* > .05).

## DISCUSSION

Evidence suggests that diet plays an important role in the development of IBD. Many patients consider diet as an important trigger for relapse, and most patients change their diet and food choices at various stages of disease activity, thereby jeopardizing their nutritional status.^13^ In the present study, the relationship between disease activity scores of patients with IBD and adherence to MD was investigated. The effect of nutritional status on quality of life was also investigated. The fundamental finding in the present study was that UC patients with low adherence to MD had higher disease activity scores. In a similar study, it was found that active Crohn’s patients had lower adherence to MD than inactive patients. However, this relationship between adherence to MD and disease activity was not observed in the UC group.^23^ Khalili et al^13^ showed that greater adherence to MD was associated with a lower risk of late-onset CD.

The relationship between MD and intestinal inflammation is explained by several mechanisms. First, adherence to MD is characterized by a high intake of vitamins, minerals, and antioxidants. Previous studies have shown that serum vitamin, mineral, and antioxidant levels are particularly lower in patients with active IBD. In addition, there is evidence to support the beneficial role of vitamins A, D, E, K, and C in influencing immunological responses in IBD.^24^ Second, adherence to MD is associated with a useful microbiome profile in healthy individuals and the production of short-chain fatty acids, which are thought to have a preventive role in IBD.^25^ However, it has been observed that the Western diet, characterized by a high intake of fats, sugars, red meat, and processed nutrients, triggers microbial dysbiosis and inflammation.^26^ A study on this subject reported that adherence to MD in patients with UC after pouch surgery was associated with reduced calprotectin levels. For this reason, MD is thought to have an important role in changing the intestinal inflammatory status in IBD. In addition to these mechanisms, it is also observed that the compound effect of the MD is larger than the individual effect of each nutrient.^27^ The limited number of studies on IBD makes it difficult to compare the differences between CD and UC patients with the current literature.

Results of the present study demonstrated that the patient’s quality of life was affected by disease activity scores and quality of life related to emotional problems, mental health, and overall health perception increased in patients with UC as the increased MD adherence. Severe symptoms and treatment regimens of IBD patients have significant detrimental effects on their lives and health-related quality of life.^28^ Current data show that IBD activity indices are associated with quality of life and that patients in the active period have a worse quality of life compared to patients in remission.^29^ In addition, Papada et al^14^ showed that high MD scores improved the quality of life in Crohn’s patients. Lo et al^29^ showed that a healthy lifestyle, including adherence to MD, was significantly correlated with decreased mortality in IBD.^29^ In general, MD is thought to have a high potential as a therapeutic and preventive tool for IBD.^30^

Despite the important findings, this study has certain limitations, such as the relatively small sample size and observational design of the study. Another limitation is that both CDAI and Mayo Clinic scores give a non-comprehensive assessment of disease activity, although they are the most widely used scoring systems by the scientific community. In addition, the lack of routine laboratory parameters that can provide information about disease activity is another limitation of our study.

## CONCLUSION

In patients with IBD, nutritional counseling and recommendations are effective in the management of the disease. In the present study, greater adherence to the MD had a significant protective effect against UC, with an increase in quality of life and decreased levels of disease activity. These findings support the recent promising evidence regarding the benefits of MD in patients with IBD. However, further prospective studies are needed to investigate the potential use of MD in the management of IBD. The present study evaluating adherence to MD in patients with IBD will hopefully guide further clinical trials in this patient population.

## Figures and Tables

**Table 1.** General Characteristics of the Patients (n = 83)

Characteristics	
Age (year)	37.4 ± 11.8
Weight (kg)	70 (42-125)
Gender, n (%)	
Female	50 (60.2)
Male	33 (39.8)
Body mass index (kg/m^2^)	26.1 ± 4.9
IBD diagnosis, n (%)	
Ulcerative colitis	38 (45.8)
Crohn’s disease	45 (54.2)
Duration of IBD diagnosis, n (%)	
1-4 years	44 (53.1)
5-10 years	39 (46.9)
Smoking, n (%)	
Yes	25 (30.1)
No	58 (69.9)
Alcohol use, n (%)	
Yes	4 (4.8)
No	79 (95.2)
Physical activity status, n (%)	
Yes	45 (54.2)
No	38 (45.8)
Crohn’s Disease Activity Index score	134 (7-416)
Crohn’s Disease Activity class, n (%)	
Remission	27 (60.0)
Mild	9 (20.0)
Moderate	9 (20.0)
Ulcerative Colitis Mayo Clinic score	5 (1-12)
Ulcerative Colitis Disease Activity class, n (%)	
Remission	5 (13.2)
Mild	18 (47.4)
Moderate	12 (31.6)
Severe	3 (7.9)
Mediterranean Diet Adherence score	7 (1-12)
Mediterranean Diet Adherence class, n (%)	
Low adherence	38 (45.8)
Acceptable adherence	27 (32.5)
High adherence	18 (21.7)

IBD, inflammatory bowel disease.

The normally distributed data are presented as mean ± standard deviation and non-normally distributed data as median (minimum-maximum).

**Table 2.** Evaluation of Mediterranean Diet Adherence Scale According to Body Mass Index and Disease Activity Scores

	Mediterranean Diet Commitment Scale			*P*
	Low Adherence (n = 28)	Acceptable Adherence (n = 13)	High Adherence (n = 7)	
Ulcerative Colitis Mayo Clinic score	7.0 (3.0-12.0)	3 (1.0-10.0)	3.0 (1.0-9.0)	**.018**
Crohn’s Disease Activity Index score	138.0 (7.0-349.0)	145.0 (28.0-416.0)	126.0 (14.0-339.0)	.425
Body Mass Index	25.1 (16.4-40.4)	27.2 (18.4-36.9)	25.1 (20.0-31.8)	.966

The non-normally distributed data are shown as median (minimum-maximum).

**Table 3.** Relationship Between Crohn’s Disease Activity Index Score, Mayo Clinic Score, and SF-36 Sub-Dimensions

	Crohn’s Disease Activity Index Score		Ulcerative Colitis Mayo Clinic Score	
	*R*	*P*	*R*	*P*
Physical function	−0.155	.146	−0.075	.538
Role limitations due to physical functions	−0.290	**.011**	−0.324	**.014**
Role limitations due to emotional problems	−0.195	.088	−0.290	**.026**
Energy/vitality	−0.152	.151	−0.332	**.006**
Mental health	−0.155	.141	−0.285	**.018**
Social function	−0.208	.056	−0.233	.060
Pain	−0.260	**.016**	−0.222	.070
Overall health perception	−0.283	**.008**	−0.362	**.003**

SF-36, Quality of Life Scale Short Form-36.

**Table 4.** The Relationship Between Adherence to MD and SF-36 Scores for UC and CD patients

	Crohn’s Disease (n = 45)				Ulcerative Colitis (n = 38)			
SF-36 Sub-Dimensions	Low Adherence (n = 18)	Acceptable Adherence (n = 13)	High Adherence (n = 7)	*P*	Low Adherence (n = 18)	Acceptable Adherence (n = 13)	High Adherence (n = 7)	*P*
Physical function	77.0 (20.0-100.0)	80.0 (20.0-95.0)	70.0 (10.0-95.0)	.49	72.5 (25.0-100.0)	80.0 (20.0-100.0)	80,00 (60-100)	.32
Role limitations due to physical functions	50.0 (0.0-100.0)	50.0 (0.0-100.0)	25.0 (0.0-100.0)	.76	25.0 (0.0-100.0)	75.0 (0.0-100.0)	100.0 (0.0-100.0)	.29
Role of limitations due to emotional problems	33.3 (0.0-100.0)	33.3 (0.0-100.0)	66.6 (0.0-100.0)	.52	33.3 (0.0-100.0)	66.6 (0.0-100.0)	100.0 (33.3-100.0)	**.03**
Energy/vitality	52.5 (10.0-85.0)	47.5 (20.0-80.0)	40.0 (5.0-80.0)	.94	35.0 (0.0-100.0)	50.0 (20.0-90.0)	60.9 (25.0-90.0)	.14
Mental health	54.0 (16.0-96.0)	48.0 (28.0-76.0)	58.0 (32.0-88.0)	.31	40.0 (0.0-100.0)	60.0 (23.0-92.0)	76.0 ([36.0-100.0)	**.03**
Social function	68.7 (0.0-100.0)	50.0 (35.0-100.0)	62.3 (0.0-100.0)	.96	62.5 (0.0-100.0)	62.5 (0.0-90.0)	75.0 (38.0-100.0)	.45
Pain	62.5 (38.0-100.0)	62.5 (0.0-100.0)	67.5 (10.0-100.0)	.66	56.2 (0.0-100.0)	90.0 (10.0-100.0)	90.0 (45.0-100.0)	.14
Overall health perception	47.7 (10.0-95.0)	45.0 (0.0-75.0)	30.0 (10.0-70.0)	.16	35.0 (0.0-75.0)	55.0 (25.0-90.0)	70.0 (35.0-85.0)	**.00**

CD, Crohn’s disease; MD, Mediterranean diet; SF-36, Quality of Life Scale Short Form-36; UC, ulcerative colitis.

The non-normally distributed data are shown as median (minimum-maximum).
